# ENDOSCOPIC MANAGEMENT OF MESH MIGRATION FOLLOWING HIATAL HERNIA REPAIR

**DOI:** 10.1590/0102-6720202400053e1847

**Published:** 2024-12-16

**Authors:** Bruno Costa MARTINS, Adrielma Athena Rodrigues Serrão MARTINS E SILVA, Ada Alexandrina Brom dos Santos SOARES, Ulysses RIBEIRO

**Affiliations:** 1 Universidade de São Paulo, Faculty of Medicine, Department of Gastroenterology – São Paulo (SP), Brazil;; 2 Hospital Alemão Oswaldo Cruz, Endoscopy Unit – São Paulo (SP), Brazil.

**Keywords:** Surgical mesh, Endoscopy, Hernia, Hiatal, Telas Cirúrgicas, Endoscopia, Hérnia Hiatal

## Abstract

**BACKGROUND:**

The use of mesh in the repair of large hiatal hernias is still controversial. One of the most feared adverse events related to the use of mesh is erosion into the esophageal and gastric walls.

**AIMS:**

To record the endoscopic treatment of mesh that has migrated into the gastric lumen after surgical treatment of hiatal hernia.

**METHODS:**

The technical option was to wait for the progressive migration of the mesh into the gastric lumen, monitoring with upper digestive endoscopy, with removal by traction at the best time, with the aid of foreign body forceps.

**RESULTS:**

The mesh was completely removed, and the evolution was satisfactory, without complications.

**CONCLUSIONs:**

In patients with mesh migration into the stomach who are oligosymptomatic and do not show signs of complications, endoscopic surveillance and subsequent removal of the foreign body can be successfully performed when the mesh is not adhered to the gastric wall, avoiding surgical procedures with high morbidity and mortality.

## RESUMO

**RACIONAL:**

O uso de tela em reparo de grandes hérnias hiatais ainda é controverso. Um dos eventos adversos mais temidos, relacionados com o uso da tela é a erosão para a parede esofágica e gástrica.

**OBJETIVOS:**

Registrar o tratamento endoscópico de tela migrada para o lúmen gástrico, após tratamento cirúrgico de hérnia de hiato.

**MÉTODOS:**

A opção técnica de aguardar a migração progressiva da tela para o lúmen gástrico, monitorando com endoscopia digestiva alta, com remoção por tração no melhor momento, com auxílio de uma pinça de corpo estranho.

**RESULTADOS:**

A remoção da tela foi completa, e a evolução foi satisfatória, sem complicações.

**CONCLUSÕES:**

Em pacientes com migração de tela para o interior do estômago, oligossintomáticos e que não apresentam sinais de complicação, vigilância endoscópica e posterior remoção do corpo estranho pode ser realizada com sucesso, quando a tela não aderida à parede gástrica, evitando procedimentos cirúrgicos com grande morbidade e mortalidade.

## INTRODUCTION

Hiatal hernia (HH) is prevalent in the general population, with incidence increasing with age, female gender, and body mass index^6^. It is commonly observed in patients with gastroesophageal reflux disease, affecting both the anatomy and physiology of the normal antireflux mechanism^16^.

The Society of American Gastrointestinal and Endoscopic Surgeons recommends that all symptomatic paraesophageal HHs should be repaired. Surgery may also be considered for selected cases of sliding hernia^7^. In patients with a significantly large hiatus, particularly if the crural fibers are disrupted during dissection or if the crural closure is tenuous or under tension, mesh repair may be employed to reinforce the weakened diaphragmatic crura and to close a hiatus that cannot be adequately plicated^7,22^. However, the use of mesh in large HH repairs remains controversial^2,13,14^. While some randomized trials have demonstrated reduced recurrence rates^4,8,10,11,19,20^, a meta-analysis of 7 randomized trials involving 735 patients found no significant difference in short-term and long-term recurrence rates^1^.

One of the most concerning adverse events associated with mesh repair is mesh erosion into the esophageal or gastric wall^3,5,14,17,18,22^. The optimal type of mesh and technique for this procedure are still a matter of debate.

The objective is to record the endoscopic treatment of mesh that has migrated into the gastric lumen after surgical treatment of HH.

## CASE REPORT

**Figure 1:**
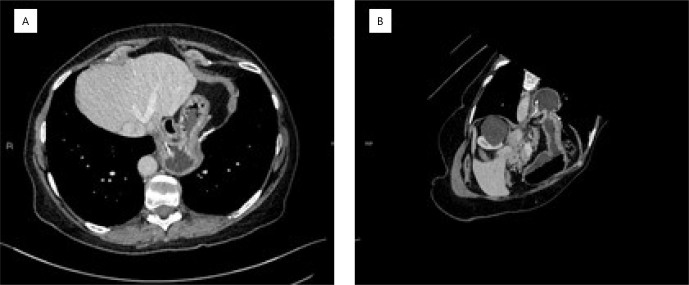
Computed tomography of the abdomen: partial mesh erosion into the gastric wall. (A) Axial section and (B) sagittal section.

A 63-year-old patient who underwent laparoscopic fundoplication and hiatoplasty with synthetic mesh for gastroesophageal reflux disease and a paraesophageal HH 12 years ago was referred to our unit for the removal of mesh that had partially migrated into the gastric lumen. The patient reported sporadic postprandial discomfort, dysphagia for solid foods, and weight loss. Computed tomography imaging identified partial migration of the mesh into the gastric lumen (Figure 1). The patient did not exhibit other complications such as obstruction, leaks, or abscesses.

**Figure 2:**
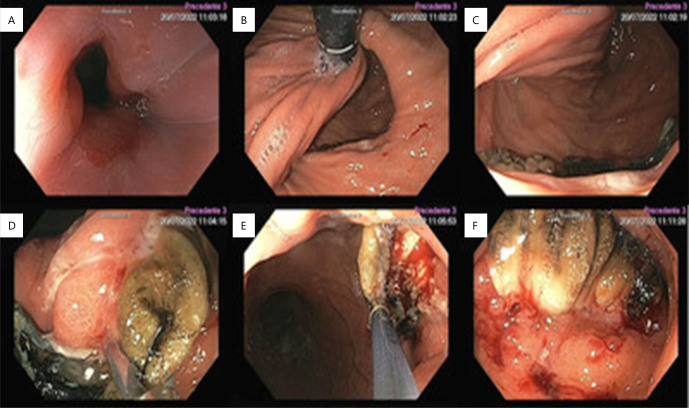
Attempt to remove in the initial upper-digestive endoscopy: (A) frontal view of the esophagogastric junction; (B and C) retroflex vision — paraesophageal hernia and partial mesh erosion; (D and E) minor bleeding occurred during removal attempt; (F) mesh firmly adhered into the gastric wall.

Initial upper-digestive endoscopy revealed a paraesophageal hernia, migration of the fundoplication to the thorax, and partial erosion of the mesh into the gastric lumen (Figure 2). During this procedure, an attempt to remove the mesh by grasping it with foreign body removal forceps and pulling it into the stomach lumen was unsuccessful. Efforts to cut the mesh with endoscopic scissors also failed. Forceful attempts to pull the mesh into the gastric lumen induced minor bleeding from the stomach wall, prompting the decision to abort the removal attempt.

**Figure 3:**
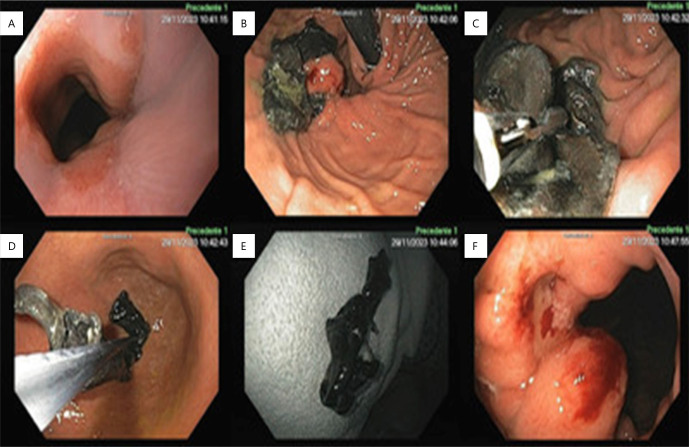
Endoscopic removal of the mesh after 16 months: (A) esophagogastric junction; (B) retroflex vision — paraesophageal hernia and mesh almost free of the stomach wall; (C and D) retrieval by traction with grasping forceps; (E) mesh removed; and (F) ulcer in the site of mesh erosion post-removal.

Given the patient’s mild symptoms and absence of complications, the medical team opted for conservative management. Serial endoscopies every 6 months showed progressive migration of the mesh into the gastric lumen. Approximately 16 months after initial observation, complete removal of the mesh was achieved using foreign body removal forceps and gentle traction into the gastric lumen (Figure 3). Post-removal, an ulcer was noted at the site of erosion without signs of active bleeding (Figure 3F), and the patient’s symptoms were completely resolved.

## DISCUSSION

This case illustrates the successful removal of a migrated mesh, into the stomach, using an endoscopic procedure, highlighting an effective management strategy for a rare but significant complication following HH repair.

The reported incidence of mesh complications in the literature ranges from 10% to 20%^12,13,15,17^, but the true incidence of mesh erosion and migration after HH repair may be underestimated^12,15^ with documented cases likely representing only a small portion of the total^9^. One reason for this is the lack of long-term follow-up in some studies as this complication can occur several years after the initial surgery^2^. Mesh erosion can result in severe morbidity and sometimes requires complex organ resection^9,17,18,21,22^. Synthetic meshes are more frequently implicated in erosions, which can occur anytime from 2 weeks to 20 years post-surgery, although the majority happen within the first 5 years^17^. One study found that 79% of mesh erosion cases occurred within 2 years following the initial HH repair^9^. The most common sites of erosion are the esophagus (50%), stomach (25%), and gastroesophageal junction (23%), with rare cases involving the bronchus, aorta, and ventricle^9,17^. In a series involving 28 patients with mesh-related complications, symptoms such as dysphagia, heartburn, chest pain, fever, epigastric pain, and weight loss were observed^18^. Of these patients, 23 required surgical intervention: six underwent esophagectomy, two had partial gastrectomy, and one had a total gastrectomy. Unfortunately, two patients died 3 months postoperatively from unknown causes, one developed severe gastroparesis, and five became dependent on tube feeding^18^.

In the present case, the patient experienced postprandial discomfort, dysphagia for solid foods, and weight loss. The mesh, composed of polypropylene, eroded into the stomach lumen 10 years after the initial surgery. The erosion occurred through the posterior wall of the stomach, likely due to a foreign body reaction.

Different treatment modalities for mesh migration have been documented, including endoscopic retrieval (15.7%), laparoscopic removal (11.8%), and surgical removal (19.6%)^15^. More invasive procedures such as distal esophageal resection and gastric resection were required in 19.6 and 5.9% of cases, respectively^9^. In this case, initial attempts to remove the mesh endoscopically with forceps and scissors failed due to the presence of firmly adherent and fibrous tissue. The team decided to wait until the mesh was less adherent, allowing for complete retrieval.

Endoscopic retrieval is feasible when the mesh is nearly free within the esophageal or gastric lumen and should be considered the first-line treatment, with a success rate of 15.7%^13,15^. This approach can be accomplished in single or multiple endoscopic attempts, potentially avoiding the need for more invasive laparoscopic or open surgical procedures^1^.

Two major concerns with endoscopic mesh removal are bleeding and perforation. In this case, the initial attempt was aborted due to the risk of bleeding or gastric laceration in a minimally symptomatic patient, which would necessitate urgent surgical intervention. Eventually, with careful monitoring and timely intervention, complete endoscopic mesh retrieval was achieved, resulting in symptom resolution and avoidance of surgery.

## CONCLUSIONS

In conclusion, in patients with gastric-lumen mesh erosion, who exhibit few symptoms and no signs of complications such as obstruction, leaks, or abscesses, monitoring and endoscopic removal can be performed when observed that the mesh is found to be almost free or completely free of the stomach wall, avoiding procedures with greater morbidity and mortality. A case-by-case strategy is necessary for such rare complications.
